# The potential biomarkers in predicting pathologic response of breast cancer to three different chemotherapy regimens: a case control study

**DOI:** 10.1186/1471-2407-9-226

**Published:** 2009-07-11

**Authors:** Linbo Wang, Zhinong Jiang, Meihua Sui, Jianguo Shen, Chaoyang Xu, Weimin Fan

**Affiliations:** 1 Department of Surgical Oncology, Sir Run Run Shaw Hospital, Zhejiang University College of Medicine, Hangzhou, PR China; 2 Department of Pathology and Laboratory Medicine, Medical University of South Carolina, Charleston, South Carolina, USA

## Abstract

**Background:**

Preoperative chemotherapy (PCT) has become the standard of care in locally advanced breast cancer. The identification of patient-specific tumor characteristics that can improve the ability to predict response to therapy would help optimize treatment, improve treatment outcomes, and avoid unnecessary exposure to potential toxicities. This study is to determine whether selected biomarkers could predict pathologic response (PR) of breast tumors to three different PCT regimens, and to identify a subset of patients who would benefit from a given type of treatment.

**Methods:**

118 patients with primary breast tumor were identified and three PCT regimens including DEC (docetaxel+epirubicin+cyclophosphamide), VFC (vinorelbine/vincristine+5-fluorouracil+cyclophosphamide) and EFC (epirubicin+5-fluorouracil+cyclophosphamide) were investigated. Expression of steroid receptors, HER2, P-gp, MRP, GST-pi and Topo-II was evaluated by immunohistochemical scoring on tumor tissues obtained before and after PCT. The PR of breast carcinoma was graded according to Sataloff's classification. Chi square test, logistic regression and Cochran-Mantel-Haenszel assay were performed to determine the association between biomarkers and PR, as well as the effectiveness of each regimen on induction of PR.

**Results:**

There was a clear-cut correlation between the expression of ER and decreased PR to PCT in all three different regimens (*p *< 0.05). HER2 expression is significantly associated with increased PR in DEC regimen (*p *< 0.05), but not predictive for PR in EFC and VFC groups. No significant correlation was found between biomarkers PgR, Topo-II, P-gp, MRP or GST-pi and PR to any tested PCT regimen. After adjusted by a stratification variable of ER or HER2, DEC regimen was more effective in inducing PR in comparison with VFC and EFC regimens.

**Conclusion:**

ER is an independent predictive factor for PR to PCT regimens including DEC, VFC and EFC in primary breast tumors, while HER2 is only predictive for DEC regimen. Expression of PgR, Topo-II, P-gp, MRP and GST-pi are not predictive for PR to any PCT regimens investigated. Results obtained in this clinical study may be helpful for the selection of appropriate treatments for breast cancer patients.

## Background

Chemotherapy is used in both inoperable and operable breast tumors, to promote tumor shrinkage and to render these tumors treatable by radical mastectomy or radiotherapy, or with the objective of down staging the tumor so that breast-conserving surgery could become a viable alternative to radical mastectomy. In recent years, preoperative chemotherapy has become the standard of care in locally advanced breast cancers [1,2]. Previous clinical trials have shown that preoperative chemotherapy allows regression of the tumor in order to avoid mastectomy and to treat clinically undetectable micrometastatic disease. In addition, preoperative chemotherapy permits the assessment of response of the primary tumor to a particular chemotherapy regimen and provides an early opportunity to alter the agents if the tumor appears clinically resistant.

Although several different classes of chemotherapeutic drugs are applied in the preoperative setting include taxanes, anthracyclines and *vinca *alkaloids, *etc*., there are currently no standard regimens or protocols for preoperative chemotherapy in breast cancer patients [2]. On the other hand, a significant proportion of breast tumors are resistant to chemotherapeutic agents. As a result, drug resistance has become the major cause of cancer chemotherapy failure and is largely responsible for breast cancer mortality. Therefore, the identification of patient-specific tumor characteristics that can improve the ability to predict response to therapy would help optimize treatment, improve treatment outcomes, and avoid unnecessary exposure to potential toxicities.

However, although the factors predicting response of breast tumors to hormonal therapy are known [3-6], relatively few studies to elucidate factors influencing the response of patients to treatment of combination chemotherapy have been undertaken. Previous studies on the response to chemotherapy in breast cancer either focused on single chemotherapy regimen or single class of anticancer drugs [7-9], or the combined treatment of chemotherapy and hormonal therapy [10,11], which may provide limited information or blunt the effectiveness of chemotherapy alone. Thus, more clinical studies investigating the potential predictive or prognostic biomarkers for breast cancer chemotherapy are needed.

Patients who exhibited pathologic complete response (pCR) to chemotherapy showed better progression-free survival and overall survival compared to those with residual tumor [12,13]. Therefore, pCR is now recognized as an independent prognostic factor of patients with breast carcinoma. To assess whether the selected biomarkers may play a role in predicting pathologic response of primary breast tumors to preoperative chemotherapy, and to identify a subset of patients who would benefit from a given type of preoperative treatment, 118 patients with operable breast cancer treated with three different preoperative chemotherapy regimens have been evaluated in the present study. These patients with primary breast tumors were subjected to three different chemotherapy regimens before surgery, including DEC (docetaxel+epirubicin+cyclophosphamide), VFC (vinorelbine/vincristine+5-fluorouracil+cyclophosphamide) and EFC (epirubicin+5-fluorouracil+cyclophosphamide). The molecular makers measured in this study include estrogen receptor (ER), progesterone receptor (PgR), human epidermal growth factor receptor 2 (HER2), P-glycoprotein (P-gp), multidrug resistance-related protein (MRP), glutathione S-transferase pi (GST-pi) and topoisomerase-II (Topo-II), based on their potential roles in modulating tumor biology and sensitivity or resistance to anticancer treatments [14-16].

## Methods

### Patient selection

One hundred and eighteen consecutive breast cancer patients presented at the Zhejiang University Sir Run Run Shaw Hospital, Hangzhou, China, who received preoperative chemotherapy during the period of February 2002 to January 2007 were enrolled in this study. Patients with radiotherapy or endocrine therapy before surgery, and those whose paraffin-embedded tissue from the biopsy was insufficient to allow a pathologic diagnosis and evaluation of biomarkers, were excluded. The median age was 49 years (range, 30–84 years). Thirty-eight patients (32%) were postmenopausal. The distribution of cases according to tumor size, clinical node status, and menopausal status is shown in Table 1. This study was approved by the ethical committee of Sir Run Run Shaw Hospital. Informed consents from patients were obtained for the use of paraffin-embedded tumor tissue.

**Table 1:** Patient characteristics (n = 118)

Characteristic	Value
Age	
Median	49
Range	30–84
Menopausal Status	
Premenopausal	80 (68%)
Postmenopausal	38 (32%)
Histologic Type	
Ductal Carcinoma	100 (84.7%)
Lobular Carcinoma	6 (5.1%)
Unclassified	12 (10.2%)
Clinical Primary Tumor	
T1	19 (16.1%)
T2	19 (16.1%)
T3	74 (62.7%)
T4	6 (5.1%)
Chemotherapeutic Regimens*	
DEC	58 (49.2%)
EFC	37 (31.4%)
VFC	23 (19.5%)

* D docetaxel, E epirubicin, C cyclophosphamide, F 5-Fluorouracil, V vinorelbine or vincristine

### Treatment plan

Preoperative chemotherapy was started within 1 or 2 days of diagnosis. Fifty-eight patients were treated with DEC regimen containing 75 mg/m^2 ^docetaxel *i.v*. on day 1, 60 mg/m^2 ^epirubicin *i.v*. on day 1 and 500 mg/m^2 ^cyclophosphamide *i.v*. on day 1 every three weeks for 2 cycles. Thirty-seven patients received EFC regimen containing epirubicin 60 (or 70) mg/m^2 ^*i.v*. on days 1 and 8, 5-fluorouracil 500 mg/m^2 ^*i.v*. on day 1 and cyclophosphamide 500 mg/m^2 ^*i.v*. on day 1 every three weeks for 2 cycles. Twenty-three patients were treated with VFC regimen containing vinorelbine 25 mg/m^2 ^*i.v*. or vincristine 1.3 mg/m^2 ^*i.v*. on day 1, cyclophosphamide 375 mg/m^2 ^*i.v*. on days 2, 4 and 6, and 5-fluorouracil 375 mg/m^2 ^*i.v*. on days 1, 3 and 5 every three weeks for 2 cycles. Table 2 shows the detailed employment of the above preoperative chemotherapy regimens in patients with primary breast tumors. Breast cancer surgery was performed after 2 weeks of the last cycle of chemotherapy.

**Table 2:** Preoperative chemotherapy regimens employed in patients

Biomarker	DEC (n = 58)	EFC (n = 37)	VFC (n = 23)	All Patients (n = 118)
ER				
negative	28	10	14	52
positive	30	27	9	66
positive rate(%)	51.7	73.0	39.1	55.9
PR				
negative	31	19	12	62
positive	27	18	11	56
positive rate(%)	46.6	48.6	47.8	47.5
HER2				
negative	24	16	9	49
positive	34	21	14	69
positive rate(%)	58.6	56.8	60.9	58.5
P-gp				
negative	44	30	19	93
positive	14	7	4	25
positive rate(%)	24.1	18.9	17.4	21.2
MRP				
negative	26	13	9	48
positive	32	24	14	70
positive rate(%)	55.2	64.9	60.9	59.3
GST-pi				
negative	35	23	13	71
positive	23	14	10	47
positive rate(%)	39.7	37.8	43.5	39.8
Topo-II				
negative	23	14	7	44
positive	35	23	16	74
positive rate(%)	60.3	62.2	69.6	62.7

* D docetaxel, E epirubicin, C cyclophosphamide, F 5-Fluorouracil, V vinorelbine or vincristine

### Assessment of pathologic response

Tumor tissues were obtained 2–3 days prior to chemotherapy *via *core biopsy or during the surgery. Tumor samples obtained before and after chemotherapy were fixed with 10% buffered-formalin, embedded in paraffin and stained with hematoxylin and eosin. The pathologic response of breast carcinomas was graded according to Sataloff's classification (primary site response classification) [17]. Given the nature of their results and using the same guidelines, we re-named the four categories in Sataloff's classification into two groups: Group I as responders (major pathologic response) and Group II as non-responders (minor or no response), both of which contain two subgroups. Therefore, patients were divided into the following four groups: Group Ia, total or near total therapeutic effect; Group Ib, subjectively more than 50% therapeutic effect but less than total or near total; Group IIa, less than 50% therapeutic effect, but effect evident; and Group IIb, no therapeutic effect. Pathologic response rate (pRR) was defined as a ratio of responders to total number of tumors (responders plus non-responders).

### Immunohistochemical assay

Immunohistochemical staining was completed in the pathology laboratory of Sir Run Run Shaw Hospital. The sections cut at 4–6 μm were deparaffinized in xylene and rehydrated in a series of graded ethanol. The slides were immersed in 10 mM citrate buffer (pH 6.0) and boiled for 15 minutes in a pressure cooker with the lid on for antigen retrieval. The slides were let cool down in the box at room temperature for 20 min and then washed with phosphate buffered saline (PBS). Endogenous peroxidase was blocked with 0.3% H_2_O_2 _in methanol for 10 min, and nonspecific binding was blocked by preincubation with 2% fetal calf serum in PBS with 0.1% sodium azide for 30 minutes [18].

The panel of markers detected by IHC was as followings: ER (Neomarker, rabbit-mAb, clone sp1), PgR (Lab Vision, rabbit-mAb, clone sp2), HER2 (Lab Vision, rabbit-mAb, clone sp3), P-gp (ZSGB-Bio, mouse-mAb, clone c219), MRP (GeneTech, mouse-mAb, clone 33A6), GST-pi (Dako, rabbit-polyAb), Topo-II (Dako, mouse-mAb, clone KI-S1). After washing three times in PBS, the slides were incubated with the EnVision-HRP complex (Dako, Carpinteria, CA) for 60 min. Finally, the slides were visualized with diaminobenzidine (Dako) and counterstained with hematoxylin. For substitute negative controls, the primary antibody was replaced with PBS. Positive controls included breast or colonic carcinomas known to exhibit high levels of each marker.

### Scoring of immunohistochemical assay

Immunohistochemical analysis reported in this study was carried out in a single laboratory. All slides were reviewed and scored independently by two pathologists without knowledge of the demographic or treatment response information. ER, PgR and Topo-II were scored as positive when at least 10% of the carcinoma cell nuclei were immunoreactive [7]. The intensity of membrane staining of HER2 was evaluated according to the criteria set forth by the DAKO Hercep Test: score 0 = no or up to 10% membrane staining; score 1+ = partial and/or faint membrane staining in more than 10% of tumor cells; score 2+ = weak to moderate complete membrane staining in more than 10% of tumor cells; and score 3+ = strong complete membrane staining in more than 10% of tumor cells [7]. Ascore of 0 and 1+ for HER2 was considered negative, whereas 2+ and 3+were considered positive. We defined ER negative, PR negative and HER2 negative as triple-negative phenotype, regardless of the expression of other biomarkers [19]. Other combinations of ER, PgR and HER2 expression was considered non-triple negative phenotype. The evaluation criteria for P-gp, MRP and GST-pi expression were interpreted based on the percentage of positive tumor cells: score 0 = no staining; score 1+ = up to 25% staining; score 2+ = 25–50% staining; and score 3+ = more than 50% staining of tumor cells [20]. Ascore of 0 was considered negative, and all other scores were considered positive.

### Statistical analysis

Descriptive studies were performed with SPSS 15.0 for windows [10,11]. Chi square test (or Fisher's exact test) and logistic regression were performed in univariate and multivariate analysis, respectively, to determine the relationship between expression of biomarkers and pathologic response. Chi square test was also used for association between different biomarkers. The relationship between different chemotherapeutic regimen and pathologic response was assessed with Cochran-Mantel-Haenszel chi-square test adjusted for the effect of a stratification variable ER. A *p *value of < 0.05 was considered significant. Two-sided statistical tests were used in all the above analyses.

## Results

### Response to preoperative chemotherapy

The pathologic responses were evaluated in all 118 patients at the end of two cycles of preoperative chemotherapy administration. According to the criteria defined above, 26 of 118 (22.0%) patients had Ia (total/near total therapeutic effect); 14 of 118 (11.9%) patients had Ib (subjectively >50% therapeutic effect but <total/near total therapeutic effect); 23 of 118 (19.5%) patients had IIa (<50% therapeutic effect); and 55 of 118 (46.6%) patients had IIb (no therapeutic effect). For further analysis, patients treated with each combination chemotherapeutic regimen were divided into two categories based on the response to treatment: responders (Ia + Ib) and nonresponders (IIa + IIb). There were 25 responders of 58 (43.1%) in DEC regimen group, 6 responders of 37 (16.2%) in EFC regimen, and 9 responders of 23 (39.1%) in VFC regimen group.

### Evaluation of immunostaining and the correlation among biomarkers

Expression of biomarkers was immunohistochemically determined in biopsy specimens obtained before preoperative chemotherapy. Positive ER, PgR and HER2 immunostainings were noted in 55.9%, 47.5% and 58.5% of the specimens, respectively. P-gp, MRP, GST-pi and Topo-II positive staining were observed in 21.1%, 60.2%, 39.8% and 62.7% of tumors, respectively. Fifteen of all 118 specimens presented triple negative phenotype (negative ER, PgR and HER2). As shown in Table 3, statistically significant correlations on expression of certain biomarkers were observed by using Chi square test. In brief, a strong direct correlation was found between ER and PgR receptor status. ER negative tumors were usually PgR negative, and only 6 tumors showed PgR positive in 52 ER negative tumors. In contrast, ER expression was inversely correlated with HER2, GST-pi and Topo-II. For example, 71.2% of ER negative tumors showed HER2 positive. In addition, significantly direct correlation was also observed between HER2 and Topo-II, as well as between GST-pi and MRP (see Table 3).

**Table 3:** Statistically significant correlations between biomarkers

Biomarkers Evaluated	R Value	*p *Value**
ER to PR	0.638	0.000
ER to HER2	-0.228	0.013
ER to GST-pi	-0.254	0.005
ER to Topo-II	-0.261	0.004
GST-pi to MRP	0.251	0.006
HER2 to Topo-II	0.204	0.027

**p < 0.05 is considered statistically significant

### Correlation between expression of biological markers and pathologic response

Table 4 summarizes the results of univariate analyses for the relationship between pathologic response and biomarkers. Moreover, the multivariate analysis was made by using the stepwise forward logistic regression model for the co-variables including ER, PgR, HER2, P-gp, MRP, GST-pi, Topo-II and three different chemotherapeutic regimens. The statistically significant independent factor in multivariate analysis was listed in Table 5.

**Table 4:** Univariate analysis for pRR and biomarkers^#^

Biomarker	DEC n = 58		EFC n = 37		VFC n = 23	
	
	pRR (%)	p**	pRR (%)	p**	pRR (%)	p**
ER						
negative	67.9	0.000**	50.0	0.003**	57.1	0.040**
positive	20.0		3.7		11.1	
PR						
negative	58.1	0.014**	26.3	0.180	58.3	0.089
positive	25.9		5.6		18.2	
HER2						
negative	16.7	0.001**	25.0	0.371	55.6	0.383
positive	61.8		16.2		28.6	
P-gp						
negative	38.6	0.223	13.3	0.315	42.1	1.000
positive	57.1		28.6		25.0	
MRP						
negative	32.0	0.137	7.7	0.394	22.2	0.228
positive	51.5		20.8		50.0	
GST-pi						
negative	42.9	0.963	8.7	0.173	30.8	0.417
positive	43.5		28.6		50.0	
Topo-II						
negative	26.1	0.034**	6.7	0.368	42.9	1.000
positive	54.3		22.7		37.5	

^# ^pRR: pathologic response rate

* D docetaxel, E epirubicin, C cyclophosphamide, F 5-Fluorouracil, V vinorelbine or vincristine

**p < 0.05 is considered statistically significant

**Table 5:** Multivariate analysis for pRR and biomarkers^#^

Treatment*	Independent Factor	OR	*p *Value**
DEC (n = 58)	ER	5.2	< 0.001
DEC (n = 58)	HER2	0.2	0.026
EFC (n = 37)	ER	26.0	0.007
VFC (n = 23)	ER	10.7	0.047
All patients (n = 118)	ER	11.6	< 0.001

^# ^pRR: pathologic response rate

* D docetaxel, E epirubicin, C cyclophosphamide, F 5-Fluorouracil, V vinorelbine or vincristine

**p < 0.05 is considered statistically significant

In univariate analysis and logistic regression analysis (Tables 4 and 5), there was a clear-cut correlation between the expression of ER and decreased pathologic response to preoperative chemotherapy in all three different regimens (*p *< 0.05). PgR was correlated with pathologic response to DEC regimen but not other two regimens in univariate analysis. However, no significant correlation between PgR and any chemotherapy regimen was found in multivariate analysis (*p *> 0.05). HER2 expression was significantly associated with increased pathologic response rate in breast tumors treated with DEC regimen in both univariate and multivariate analyses, suggesting that HER2 was an independent predictive factor for DEC treatment (*p *= 0.026). However, no significant predictive effect of HER2 was found in EFC and VFC regimens, although HER2 negative tumors showed slightly higher pathologic response rate than positive tumors (*p *> 0.05, see Table 4). Tumors with triple negative phenotype (negative ER, PgR and HER2) achieved significantly higher pathologic response rate than non-triple negative phenotype (60.0% *versus *30.1%, *p *= 0.022). Interestingly, in ER negative tumors, the pathologic response rate of triple negative tumors was similar with non-triple negative tumors (60.0% versus 62.2%, *p *= 0.885).

A correlation between Topo-II expression and greater pathologic response to preoperative chemotherapy in DEC regimen group was observed in univariate analysis (*p *= 0.034) but not in multivariate analysis (*p *= 0.202). No statistical significance between Topo-II expression and pathologic response was achieved in EFC and VFC regimens (*p *= 0.368 and 1.000, respectively) in univariate analysis. Moreover, no significant correlation was found between biomarkers P-gp, MRP or GST-pi and pathologic response to preoperative treatment of any chemotherapy regimen investigated in this study.

### Histological features of the primary breast tumors with different estrogen receptor status to preoperative chemotherapy

**Figure 1:**
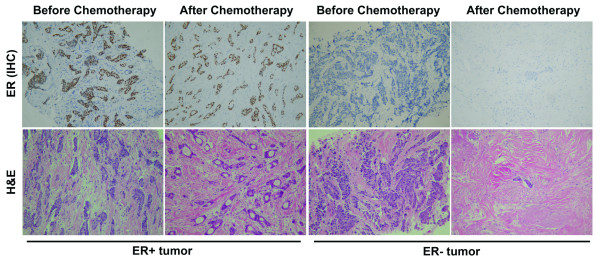
**Immunohistochemical staining with ER (upper panel) and H&E staining (lower panel)**. Stainings were performed on formalin-fixed, paraffin-embedded tissue sections of invasive ductal breast carcinoma, and photographed before and after preoperative chemotherapy treatment of EFC regimen, by using a Zeiss Axioskop40 Microscope and a Nikon E4500 camera. In ER+ tumor sample (left), tumor cells arranged in glands and clusters still showed active growth after the EFC chemotherapy treatment. Both core needle biopsy specimen and surgical specimen showed diffuse nuclear staining for estrogen receptor (diaminobenzidine chromogen). However, in ER- tumor sample (right), only a small nest of tumor cells remained survival after EFC treatment, with prominent stromal fibrosis and hyalinization. ER staining was negative in both biopsy and surgical specimens. Data are representative of at least three separate experiments. *Magnification*:×*100*.

The histological effects of preoperative chemotherapy in primary breast tumors with different ER status were evaluated by comparing biopsy and surgical samples taken before and after chemotherapy. Tumor samples were fixed in formalin for hematoxylin and eosin (H&E) staining and immunohistochemical staining with anti-ERα antibody using standard protocols. Various pathological changes were observed after preoperative chemotherapy, which mainly included coagulative necrosis of tumor tissue, fibrosis/hyalinization, as well as mixed inflammatory infiltrate. Chemotherapy could also induce cytological changes in tumor cells in cytoplasm and/or nuclear. For example, the cytoplasm of tumor cells was either intensely eosinophilic or clear with a vacuolated or foamy appearance. Enlarged or bizarre nuclear with clumped chromatin may be recognized. Furthermore, the volume of residual tumor varied based on different chemotherapeutic effects. In our study, most ER-negative breast tumors showed significant better pathologic response to chemotherapy than ER-positive tumors. As shown in Fig. 1, there were still many survival tumor cells with ER positivity after the preoperative chemotherapy of EFC regimen in ER-positive primary breast tumors. However, the ER-negative primary tumor exhibited significantly improved pathologic response to EFC treatment, which was demonstrated by the dramatically reduced volume of residual tumor, as well as the remarkable fibrosis and hyalinization of tissue after the treatment.

### Correlation between chemotherapy regimen and pathologic responses

As described above, our results indicate that ER is an independent predictive factor for all three different chemotherapy regimens investigated in this study. Because the positive rates of ER in tumors treated with DEC and VFC regimens (51.7% in DEC group and 39.1% in VFC group) were obviously lower than that in EFC group (73.0%), Cochran-Mantel-Haenszel chi-square test was utilized to compare the discrepancy of pathologic response to different chemotherapy regimens, adjusted by the effect of the stratification variable ER. After adjusted by the effect of ER, our results indicate that breast tumors treated with DEC regimen achieved higher pathologic response than tumors treated with EFC or VFC regimen (DEC *versus *EFC, *p *= 0.044; DEC *versus *VFC, *p *= 0.030), while the pathologic response rate induced by VFC regimen was similar to that induced by EFC regimen (*p *= 0.495). The difference on disparity of pathologic response rate between the above two groups appears more remarkable in ER-positive tumors than in ER-negative tumors. For example, only one of total twenty six ER-positive tumors achieved major pathologic response by treatment of EFC regimen (pRR = 3.7%), and in six of thirty ER-positive tumors in DEC group achieved pathologic response (pRR = 20%), although the difference did not reach statistical significance (*p *= 0.105). While in ER-negative tumors, the difference on pathologic response between these two regimens was relatively minor (67.9% in DEC regimen *versus *50% in EFC regimen, *p *= 0.449). Moreover, after adjusted by the effect of stratification variable HER2, our data indicated that tumors treated with DEC regimen achieved significantly higher pathologic response than tumors treated with other two regimens (DEC *versus *EFC, *p *= 0.004; DEC *versus *VFC, *p *= 0.03) in HER2 positive tumors, while the pathologic response rates induced by VFC or EFC regimens were similar (*p *= 0.191). However, this superiority of DEC regimen to other two regimens was not observed in HER2 negative tumors.

## Discussion

The future of cancer treatment lies in tailoring regimens to individual patients by identifying response predictors and developing novel agents. The preoperative chemotherapy is an ideal clinical setting in which to validate the relationship between tumor molecular profiling and treatment outcomes, and to optimize therapies based on observed effects on individual tumors. As a result, the evaluation of pathologic response to preoperative chemotherapy provides an ideal platform to identify the potential predictive factors of response to anticancer drugs. The present clinical study was undertaken to determine whether the expression of ER, PgR, HER2, P-gp, MRP, GST-pi and Topo-II could affect the pathologic response of primary breast tumors to three different preoperative chemotherapy regimens including DEC, VFC and EFC. A clear definition of such factors would assist in the selection of appropriate treatments for breast cancer patients.

Steroid hormone receptors, ER and PgR, are important biological markers of breast carcinoma. Their status has an established role in determining the tumor response to hormonal therapy, but their role in predicting response to chemotherapy is less clear [3-6,14]. Previous investigations evaluating the potential correlation between estrogen receptor status and pathologic response to chemotherapy have produced contradictory data: ER-positive tumors respond either better [21], less well [22-27] or the same [28-31], when compared with ER-negative tumors. Some results can be confused by the use of chemo-endocrine therapy, where the receptor-positive population may have responded to the hormonal part of the treatment [10,11]. The close correlation between ER and PgR observed in this study may be explained by the regulation of PgR expression by estrogen and estrogen receptor [32,33]. However, although PgR was correlated with pathologic response to DEC regimen in univariate analysis (Table 4), no significant correlation between PgR and any chemotherapy regimen was found in multivariate analysis (Table 4 and 5). By using both univariate analysis and logistic regression, our data indicate that ER is an independent predictive factor of the pathologic response to all three different chemotherapy regimens investigated in this study including DEC, VFC and EFC. A clear correlation between the expression of ER and decreased pathologic response to preoperative chemotherapy of all three different regimens was observed in this study (Table 4 and 5, Fig. 1). In fact, through stable transfection of estrogen receptor alpha into ER-negative human breast cancer cells, we recently demonstrated that ER may mediate breast tumor resistance to taxanes and *vinca *alkaloids through inhibition of drug-induced apoptotic cell death [27]. Our findings that ER-negative tumors exhibited improved pathologic response to three different combination chemotherapy regimens suggest that ER may also mediate breast cancer resistance to other chemotherapeutic drugs. Our data provide strong support for the predictive value of ER and indicate that ER seems to be more sensitive than PgR in predicting pathologic response to preoperative chemotherapy with DEC, VFC or EFC regimens.

Amplification and overexpression of certain oncogenes have been associated with an aggressive natural history, poor prognosis and altered sensitivity to chemotherapy (either chemosensitization or chemoresistance). Among them, one of the most promising new markers is the Her2 gene and its product (also designated c-erbB-2, c-neu) [34]. HER2 encodes a 185-kDa transmembrane tyrosine kinase active protein, which is a component of the epidermal growth factor receptor family [34]. HER2 is over-expressed in 25–30% of breast tumors. In current study, the positive rate of HER2 was 58.5%, which was greater than expected. It has been shown that more advanced breast tumors usually have relatively higher expression level of HER2 [35]. Thus, we think the high proportion of HER2+ tumors may be related with the advanced clinical stage of breast cancer patients enrolled in this study. There is controversy about the overexperssion of HER2 in relation to response of breast tumor cells to chemotherapy [36-39], and the underlying mechanism involved in these processes have not been clearly elucidated. In the present study, both univariate and multivariate analysis indicated that high level of HER2 expression was significantly associated with increased pathologic response rate in breast tumors treated with DEC regimen, suggesting that HER2 might be an independent predictive factor for DEC treatment (Table 4). However, no significant predictive effect of HER2 was found in patients treated with EFC or VFC regimens (*p *> 0.05, see Table 4). Triple-negative breast cancer is a recent term and refers to cancers that do not express ER, PgR and HER2 receptors. Histologically, such cancers are poorly differentiated, and most fall into the basal subgroup of breast cancers, characterized by staining for basal marker (*i.e*. cytokeratin) [19]. Clinically, triple-negative cancers were characterized by an aggressive clinical history, and some reports indicate that this group of breast cancer patients was associated with poor clinical outcome [19]. Very few studies have investigated the pathologic response of triple-negative breast cancers to chemotherapy [40-42]. In the present study, we found that breast tumors with triple-negative phenotype achieved significantly higher pathologic response rate than nontriple-negative phenotype (60.0% *versus *30.1%, *p *= 0.022). Interestingly, in ER-negative tumors, the pathologic response rate of triple-negative tumors was similar with nontriple-negative tumors (60.0% *versus *62.2%, *p *= 0.885), suggesting that ER might be a more important biomarker in predicting pathologic response of breast tumors to preoperative chemotherapy than PgR and HER2.

P-gp and MRP, two ATP-dependent drug transport pumps, have been indicated to confer resistance to a number of chemotherapeutic agents such as paclitaxel, epirubicin and vinblastine [16]. The expression levels of these two transporters are 21.1% and 60.2%, respectively, in our tumor samples. A significant correlation between expression of MRP and GST-pi was observed in this study (Table 3), which is consistent with previous reports indicating that GSH is necessary for MRP-mediated cellular efflux of certain drugs and detoxification of anticancer agents involves a combined action of GSTs and MRPs [43,44]. A significant positive correlation between P-gp expression and a poor clinical response to chemotherapy was reported in some studies [45,46]. However, no statistical association between the expression of P-gp, combined MRP and GST-pi with pathologic response to any of the three preoperative chemotherapy regimens was observed in the present study and several other reports [43,44,47]. On the other hand, Topo-II is an important DNA binding enzyme that modifies DNA topology. Topo-II gene is located at chromosome band 17q12-21, close to the Her2 gene [15]. A significant proportion of breast cancers with Her2 amplification show simultaneous amplification of Topo-II [24], which coincides with the significant correlation between Topo-II and HER2 protein expression observed in this study (Table 3). Some clinical investigations have shown certain association between Topo-II and response of breast cancer to chemotherapy [48-50], but some others failed to confirm this association [51]. In our study, a statistical significance between Topo-II expression and pathologic response to preoperative chemotherapy was only observed in DEC regimen in univariate analysis (*p *= 0.034), but this significance was not confirmed in multivariate analysis (*p *= 0.202).

By using a modified method of Sataloff DM to classify the pathologic response, 40 of total 118 patients in this study were classified as responders (pRR = 22.9%). Further, we evaluated the pathologic response induced by each chemotherapeutic regimen with Cochran-Mantel-Haenszel chi-square test adjusted by ER or HER2, two independent predictive biomarkers observed in this study. Our data show that after adjusted by the effect of ER, the pathologic response rate induced by DEC regimen was significantly higher than EFC and VFC regimens, while the later two regimens did not reach statistical significance. The above difference was particularly remarkable in ER-positive breast tumors than ER-negative tumors. After adjusted by the variable HER2, DEC regimen was also most effective in inducing pathologic response in HER2 positive breast tumors. These data may suggest a potential advantage in inducing relatively high pathologic response in primary breast tumors by DEC regimen compared with EFC and VFC regimens.

## Conclusion

Our data show that ER is an independent predictive factor of pathologic response to three different preoperative chemotherapy regimens including DEC, EFC and VFC in breast tumors. ER-negative tumors exhibited significantly increased benefit from the above preoperative regimens compared to ER-positive tumors. HER2 is an independent predictive factor in tumors treated with DEC regimen, but not in tumors exposed to EFC or VFC regimens. Expression of PgR, Topo-II, P-gp, MRP and GST-pi were not predictive for pathologic response to any of the three preoperative chemotherapy regimens. Results obtained in this clinical study may provide valuable information on the potential correlation between selected biomarkers with pathologic response to preoperative chemotherapy in breast tumors, which may be helpful in the selection of appropriate treatments for breast cancer patients.

## Competing interests

The authors declare that they have no competing interests.

## Authors' contributions

LW and WF were responsible for the design of this study and overall analysis, and contributed equally to this work. ZJ and MS participated in the immunohistochemical staining, performed statistical analyses and prepared the manuscript. JS contributed to the design of this study and the interpretation of data. CX supported the scoring of immunohistochemical staining. All authors read and approved the submission of the manuscript.

## Pre-publication history

The pre-publication history for this paper can be accessed here:

http://www.biomedcentral.com/1471-2407/9/226/prepub
